# Factors influencing the effectiveness of recombinant human soluble thrombomodulin on disseminated intravascular coagulation: a retrospective study

**DOI:** 10.1186/s40780-020-00183-7

**Published:** 2020-12-02

**Authors:** Yuki Asai, Takanori Yamamoto, Daisuke Kito, Kazuya Ichikawa, Yasuharu Abe

**Affiliations:** Pharmacy, National Hospital Organization Mie Chuo Medical Center, 2158-5 Hisaimyojincho, Tsu, Mie 514-1101 Japan

**Keywords:** Recombinant human soluble thrombomodulin, Disseminated intravascular coagulation, Prothrombin time-international normalized ratio, Antithrombin III, Intensive care unit

## Abstract

**Background:**

Although recombinant human soluble thrombomodulin (rTM) has been widely used to treat disseminated intravascular coagulation (DIC) in Japan, there is no consensus regarding rTM efficacy. Therefore, if the factors influencing rTM efficacy is revealed, it may be possible to demonstrate the effectiveness of rTM by limiting the patients who use rTM. This study investigated the factors of rTM treatment which influence DIC status.

**Methods:**

This retrospective case-control study enrolled hospitalized adult patients treated with rTM from October 2010 to May 2020. Among these patients, 227 who were diagnosed with DIC according to the Japanese Association for Acute Medicine DIC scoring system were assessed. The primary endpoint was the 28-day mortality after rTM treatment. For Cox-proportional hazards model, explanatory factors determined using univariate analysis with *p* <  0.1 were used. In addition, some factors considered to affect DIC-related mortality such as age ≥ 75 years, rTM dose ≥380 U/kg, antithrombin III treatment, and diseases with a poor prognosis (sepsis, solid tumors, and trauma) were added as covariates.

**Results:**

Univariate analyses suggested that male sex (*p* = 0.029), treatment in intensive care unit (*p* = 0.061), and prothrombin time-international normalized ratio (PT-INR) (*p* <  0.001) were the factors influencing DIC-related 28-day mortality after rTM treatment. According to Cox-proportional hazard analysis, the adjusted odds ratio for DIC-related 28-day mortality in patients with PT-INR ≥ 1.67 was 2.23 (95% confidence interval: 1.451–3.433, *p* <  0.001), age ≥ 75 years was 1.57 (95% confidence interval: 1.009–2.439, *p* = 0.046), and male sex was 1.66 (95% confidence interval: 1.065–2.573, *p* = 0.025), respectively. As life-threatening bleeding events were not observed, prolonged PT-INR might directly or indirectly affect DIC-related mortality caused by rTM treatment.

**Conclusion:**

rTM treatment for DIC was less effective in male patients with PT-INR ≥ 1.67 and age ≥ 75 years.

## Background

Disseminated intravascular coagulation (DIC) is among the most common emergency conditions and is characterized by organ damage because of microvascular obstruction by excessive thrombin production [1]. Simultaneous administration of platelets and coagulation factors leads to systemic hemorrhage. Because DIC is associated with high mortality, both early diagnosis and appropriate therapy are essential to improve patient outcomes [2].

Thrombomodulin (TM) is a protein that binds with high affinity to thrombin and protein C receptor, thus performing an important role in regulating coagulation [3]. Activated protein C produced following controlled degradation of the thrombin-TM complex inhibits thrombin generation by degrading and inactivating the coagulation factors VIIIa and V [4]. Recombinant human soluble thrombomodulin (rTM), a novel anticoagulant, has been widely used to improve DIC status in Japan [5–7]. Recently, Saito et al. [5] revealed no significant differences in DIC resolution rates following rTM treatment and heparin treatment; they also demonstrated that bleeding events following rTM treatment were lower than following heparin treatment. Although rTM treatment is highly effective and safe according to many studies [5–7], a randomized, double-blind, multinational, multicenter phase 3 study (SCARLET Randomized Clinical Trial) has reported no significant differences in 28-day mortality rate following rTM therapy and that following placebo therapy [8]. Consequently, there is no consensus on the effectiveness of rTM because some factors could affect the research results. Therefore, revealing the factors influencing rTM efficacy may help in demonstrating the effectiveness of rTM by limiting the patients receiving rTM.

The aim of the present study was to determine the factors influencing DIC-related mortality in patients after rTM treatment.

## Methods

### Subjects

This single-center, retrospective case-control study was performed at the National Hospital Organization Mie Chuo Medical Center (Mie, Japan), using electronic medical records. Herein, DIC was evaluated using the diagnostic criteria specified by the Japanese Association for Acute Medicine (JAAM) DIC scoring system (Table 1) [9]. DIC was diagnosed when the JAAM DIC score exceeded 4 points. We selected 251 adult, hospitalized patients treated with rTM from October 2010 to May 2020. Among these patients, 227 patients diagnosed with DIC were enrolled.

**Table 1:** Diagnostic criteria for DIC by the JAAM scoring system [9]

			Point
SIRS score [10]	Body temperature (°C)	38≦ or ≦36	
	Respiratory rate	20/min≦	
	Heart rate	90/min≦	
	WBC (/μL)	12,000≦ or ≦4000	
		positive factor 3≦	1
PLT (× 10^4^/μL)		8≦ <12	1
		<8	3
PT-INR		1.2≦	1
FDP (μg/mL)		10≦ <25	1
		25≦	3
DIC is diagnosed when exceeded total of 4 points			

*FDP* fibrinogen degradation products, *PLT* platelet, *PT-INR* prothrombin time-international normalized ratio, *SIRS* systemic inflammatory response syndrome, *WBC* white blood cell

### Baseline characteristics

The primary outcome was 28-day mortality after rTM treatment. We classified patients into survival and death groups based on whether they survived for 28 days. Baseline clinical characteristics and laboratory data that were examined in parallel to rTM treatment (Table 2).

**Table 2:** Baseline clinical characteristics and laboratory data of patients

Factors	Survival	Death	*p* value
	*n* = 124	*n* = 103	
Sex (Male/Female)	69/55	72/31	0.029^a^
Age	77 (68.5–85.5)^e^	79 (72–87)^e^	0.702^b^
Body weight (kg)	51.3 (45.8–60.0)^e^	48 (42–59)^e^	0.322^b^
eGFR (mL/min/1.73 m^2^)	38.5 (16.4–57.3)^e^	42.5 (22.9–69.1)^e^	0.272^c^
Proportion of patients with sepsis-induced DIC (%)	96	93	0.283^a^
Proportion of patients with solid tumor-induced DIC (%)	1.5	4	0.411^a^
Proportion of patients with trauma-induced DIC (%)	2.5	3	1.000^a^
SIRS score	2 (1.0–3.0)^e^	2 (1.0–3.0)^e^	0.226^b^
FDP (μg/mL)	32.5 (19.2–64.8)^e^	41.6 (20.7–77.1)^e^	0.137^c^
PLT (×10^4^/μL)	5.6 (3.8–7.7)^e^	5.5 (3.2–7.4)^e^	0.688^c^
PT-INR	1.26 (1.17–1.44)^e^	1.44 (1.28–1.72)^e^	< 0.001^c^
Proportion of patients with warfarin treatment (%)	2.5	1.9	1.000^a^
DIC score^d^	7 (5.8–9.0)^e^	7 (6.0–9.0)^e^	0.365^b^
rTM dose (U/kg)	351 (246–381)^e^	312 (223–381)^e^	0.508^b^
Number of patients treated with ATIII	31	25	1.000^b^
Number of patients treated in ICU	63	39	0.061^a^

*eGFR* estimate glomerular filtration rate, *SIRS* systemic inflammatory response syndrome, *FDP* fibrinogen degradation products, *PLT* platelet, *PT-INR* prothrombin time-international normalized ratio, *ATIII* antithrombin III, *ICU* intensive care unit

^a^Fisher’s exact test. ^b^Student’s *t*-test. ^c^Mann–Whitney *U* test. ^d^JAAM score. ^e^Each value represents the median (25–75% percentile)

### Statistical analysis

**Fig. 1:**
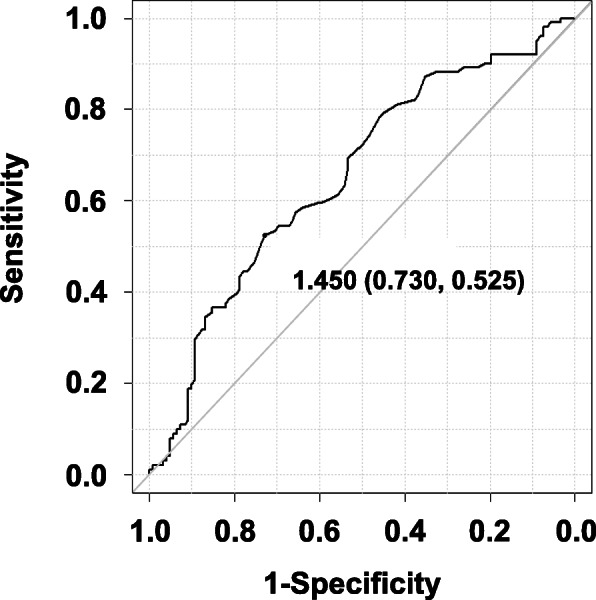
ROC curve of PT-INR for DIC-related 28-day mortality after rTM treatment

As previous studies reported that the 28-day mortality after rTM treatment was lesser than 40% [8, 11], we expected a minimum 28-day mortality rate of 40% after rTM treatment. Considering 90% power for this study and a significance level of 0.05, sample size was calculated as 182 per group. Continuous variables were compared between the survival and death groups using Student’s *t*-test or Mann–Whitney *U* test. Fisher’s exact test was conducted to compare categorical variables between the groups. The overall survival rate was calculated using Kaplan–Meier estimator and compared using the log-rank test. The missing value of PT-INR in the survival (*n* = 2) and death groups (n = 2) were replaced by the median values of each group, respectively. Multivariate analysis was conducted using the Cox-proportional hazards model. For Cox-proportional hazards model, explanatory factors determined using univariate analysis with *p* <  0.1 were used. Furthermore, some factors considered to affect DIC-related mortality including age ≥ 75 years [12], rTM dose ≥380 U/kg [13], ATIII treatment [11], and diseases with a poor prognosis (sepsis, solid tumors, and trauma) [1] were added as covariates. Moreover, the cutoff value for the extracted continuous variables calculated from receiver operating characteristic (ROC) analysis was used (Fig. 1). Statistical analyses were performed using EZR software (Saitama Medical Center, Jichi Medical University, Saitama, Japan) [14] with significance established at *p* < 0.05.

## Results

**Fig. 2:**
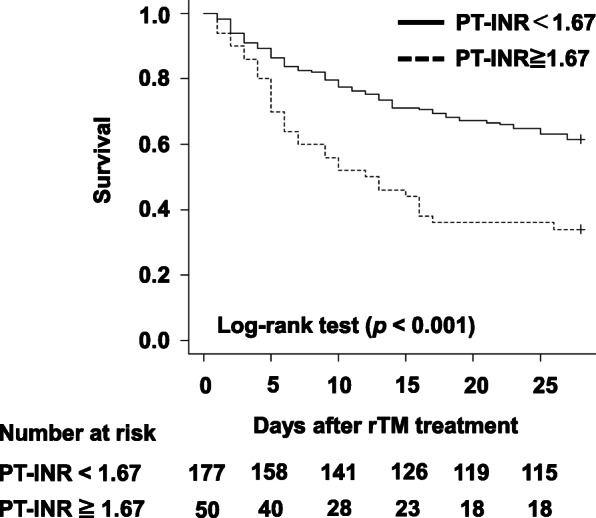
28-day survival curves after rTM in patients with PT-INR ≥ 1.67 and PT-INR < 1.67. The survival rate of patients with PT-INR < 1.67 was significantly higher than that of patients with PT-INR ≥ 1.67 (*p <* 0.001). PT-INR, prothrombin time-international normalized ratio; rTM, recombinant human soluble thrombomodulin

The proportion of patients with the subtype of diseases that caused DIC, such as sepsis, solid tumors, and trauma, was shown in Table 2, but the proportion was not statistically different between the survival and death groups. The 28-day mortality rate after rTM treatment was 45.4% (124/227 patients). Univariate analysis revealed that male sex (*p* = 0.029), treatment in ICU (*p* = 0.061), and PT-INR (*p* < 0.001) are possible factors influencing DIC-related 28-day mortality after rTM treatment (Table 2). The PT-INR cutoff value was 1.45 which corresponds to the results of the ROC analysis (sensitivity: 73%, specificity: 53%, area under the curve: 0.65) (Fig. 1). Figure 2 displays Kaplan–Meier curves for 28-day mortality after rTM treatment in patients with PT-INR < 1.67 and PT-INR ≥ 1.67. The adjusted odds ratio for 28-day mortality in patients with PT-INR ≥ 1.67 was 2.23 (95% confidence interval: 1.451–3.433, *p* < 0.001), age ≥ 75 years was 1.57 (95% confidence interval: 1.009–2.439, *p* = 0.046), and male sex was 1.66 (95% confidence interval: 1.065–2.573, *p* = 0.025) (Table 3) Life-threatening bleeding events were not observed in the mortality group.

## Discussion

The 28-day mortality rate after rTM treatment was consistent with those reported in previous studies [8, 11]. Although the DIC scores in the survival and death groups were similar, the efficacy of rTM treatment were different. Therefore, we performed Cox-proportional hazard analysis to elucidate the influential factors.

Elderly patients have reportedly higher mortality rates due to DIC [12]. In this study, we confirmed that the mortality rate was higher for patients over 75 years old than those under 75 years old, indicating that elderly patients have a high mortality rate from DIC even after rTM administration.

According to a previous study, effective blood concentration is not reached unless the dose of rTM is 380 U/kg [13]. Our study showed that the rTM dosage might not be related to efficacy for DIC-related mortality. Further studies are needed to understand the association between blood rTM concentrations and DIC resolution.

Although ROC analysis indicated that the cutoff value of PT-INR was 1.45 (Fig. 1), its clinical relevance is unknown. In the Japanese Ministry of Health and Welfare scoring system, the severity according to PT-INR < 1.25, 1.25 ≤ PT-INR < 1.67, and 1.67 ≤ PT-INR are scored as 0, 1, and 2 points, respectively, [2]. Therefore, we selected PT-INR ≥ 1.67 as the cutoff value. Cox-proportional hazard analysis demonstrated that the 28-day mortality rate in patients with PT-INR ≥ 1.67 was significantly lower than that in patients with PT-INR < 1.67 (Table 3 and Fig. 2), suggesting that PT-INR ≥ 1.67 might be a key factor influencing rTM treatment. The major adverse events occurring in patients with DIC who receive rTM are bleeding-related events [15]. Sugawara et al. [15] have suggested that rTM increases the incidence of bleeding-related adverse events, implying that rTM therapy in patients with PT-INR ≥ 1.67 increases the risk of these events. Conversely, an animal study [16] has shown that rTM does not affect the clotting time. Considering our study results, hemorrhage might not affect the 28-day mortality after rTM treatment. A study has revealed that prolonged PT-INR and FDP correlates with DIC severity [2]. Since both severe and mild cases of DIC status were randomly selected in the SCARLET study, the effectiveness of rTM was not proved conclusively [8], suggesting that the rTM treatment for mild DIC status (PT-INR ≥ 1.67) might be effective. However, the value of FDP was not different between the survival and death groups (Table 1), indicating that severe DIC status and other factors related to PT-INR affected rTM efficacy. Warfarin treatment is known to elevate PT-INR [17]. In the present study, because five subjects (three and two subjects from the survival and death groups, respectively) were treated with warfarin (Table 2), their prolonged PT-INR might be influenced by warfarin treatment. Further studies are needed to understand whether prolonged PT-INR directly or indirectly affects DIC-related mortality.

**Table 3:** Cox proportional hazard analysis of some factors of 28-day mortality following rTM treatment

Factors	Adjusted OR	95% CI	*p* value
Male	1.66	1.065–2.573	0.025
Age≧75	1.57	1.009–2.439	0.046
Sepsis-induced DIC	0.32	0.075–1.369	0.125
Solid tumor-induced DIC	0.54	0.096–3.057	0.487
Trauma-induced DIC	0.45	0.074–2.787	0.393
PT-INR≧1.67	2.23	1.451–3.433	< 0.001
rTM Dose≧380 U/kg	0.95	0.608–1.484	0.820
ATIII co-treatment	0.95	0.598–1.501	0.818
Treatment in ICU	0.91	0.595–1.380	0.645

*OR* odds ratio, *95%CI* 95% confidence interval

*ATIII* antithrombin III, *ICU* intensive care unit, *PT-INR* prothrombin time-international normalized ratio

Although male sex was extracted as a factor influencing DIC-mortality after rTM treatment (Table 3), the phenomenon of sex differences has not been reported. Estrogen, a potent steroid hormone present in high levels in females, may have great benefits in anti-inflammation and vascular protection [18]. It is speculated that differences in sex hormones can affect the reactivity of rTM, but further investigation is needed.

Our study design has several limitations. First is the lack of statistical power given the small sample size. Second, because this was a retrospective study, DIC resolution rate could not be evaluated owing to the lack of corresponding information in electronic medical records. Third, non-life-threatening bleeding events, such as gastrointestinal bleeding or intracerebral bleeding, could not be confirmed. Fourth, the underlying diseases in patients were often unknown due to the lack of corresponding information in electronic medical records. Fifth, since the sequential organ failure assessment score was measured only in ICU patients at the Mie Chuo Medical Center from 2019, organ damage was not estimated in the present study.

## Conclusions

This study reveals that PT-INR ≥ 1.67, age ≥ 75 years, and male sex may be a factor associated with DIC-related 28-day mortality after rTM treatment. Although the mechanisms underlying the increased mortality rate in patients with PT-INR ≥ 1.67 remain unknown, we proposed that rTM treatment may be less effective in such patients.

## Data Availability

All the data generated or analyzed in this study are included in the published article.

## Abbreviations

DIC: Disseminated intravascular coagulation
FDP: Fibrinogen degradation products
ICU: Intensive care unit
JAMA: Japanese association for acute medicine
PT-INR: Prothrombin time-international normalized ratio
ROC: Receiver operating characteristic
rTM: Recombinant human soluble thrombomodulin
TM: Thrombomodulin
